# Antibody–drug conjugate, GSK2857916, in relapsed/refractory multiple myeloma: an update on safety and efficacy from dose expansion phase I study

**DOI:** 10.1038/s41408-019-0196-6

**Published:** 2019-03-20

**Authors:** Suzanne Trudel, Nikoletta Lendvai, Rakesh Popat, Peter M. Voorhees, Brandi Reeves, Edward N. Libby, Paul G. Richardson, Axel Hoos, Ira Gupta, Veronique Bragulat, Zangdong He, Joanna B. Opalinska, Adam D. Cohen

**Affiliations:** 1 Princess Margaret Cancer Centre, Toronto, ON Canada; 2 Department of Medicine, Myeloma Service, Memorial Sloan Kettering Cancer Center, New York, NY USA; 3 Department of Medicine, Weill Medical College of Cornell University, New York, NY USA; 4 NIHR/University College London Hospital Clinical Research Facility, NHS Foundation Trust, London, UK; 5 Levine Cancer Institute, Atrium Health, Charlotte, NC USA; 6 Lineberger Comprehensive Cancer Center, University of North Carolina, Chapel Hill, NC USA; 7 University of Washington, Seattle, WA USA; 8 Dana-Farber Cancer Institute, Boston, MA USA; 9 GSK, Philadelphia, PA USA; 10 Abramson Cancer Center, University of Pennsylvania, Philadelphia, PA USA

**Keywords:** Drug therapy, Drug development

## Abstract

Interim analyses of a phase I study with GSK2857916, an antibody–drug conjugate against B cell maturation antigen, have previously reported a 60% overall response and 7.9 months progression-free survival in relapsed/refractory multiple myeloma (MM). We provide updated safety and efficacy results of the BMA117159 trial following an additional 14 months' follow-up. This open-label, first-in-human, phase I study was conducted at nine centres in the USA, Canada and the UK, and included adults with MM and progressive disease after stem cell transplantation, alkylators, proteasome inhibitors, and immunomodulators. In part 1, the recommended dose of 3.4 mg/kg was identified; in part 2, patients received GSK2857916 3.4 mg/kg once every 3 weeks. Selected part 2 safety/tolerability and efficacy endpoints are reported. Twenty-one (60.0%; 95% confidence interval (CI) 42.1–76.1) of 35 patients achieved partial response or better, including two stringent complete responses and three complete responses. The median progression-free survival was 12 months and median duration of response was 14.3 months. Thrombocytopenia and corneal events were commonly reported; no new safety signals were identified. GSK2857916 was well tolerated and demonstrated a rapid, deep and durable response in heavily pre-treated patients with relapsed/refractory MM, consolidating the interim analyses conclusions that GSK2857916 is a promising treatment for these patients.

## Introduction

Multiple myeloma (MM) is a plasma cell malignancy characterised by clonal proliferation of plasma cells within the bone marrow^1^. While advances have been made in the management of MM in recent years with the introduction of novel therapies such as immunomodulators and proteasome inhibitors, outcomes are poor for those with relapsed and refractory disease^2^, highlighting the need for new treatments.

B cell maturation antigen (BCMA) is a cell-surface receptor required for the survival of plasma cells^3^. BCMA is also ubiquitously expressed on MM cells^4^ and its serum levels correlate with response to therapy and overall survival in patients with MM^5^. BCMA membrane expression is universally detected in patient-derived CD138-positive myeloma cells, but not in other tissues. As such, BCMA has emerged as a very selective antigen to be targeted by novel immune-based strategies for the treatment of MM.

GSK2857916 is a humanised monoclonal anti-BCMA antibody, which is afucosylated and conjugated to the microtubule-disrupting agent monomethyl auristatin-F (MMAF)^6^. Upon binding to BCMA on the cell surface, GSK2857916 is rapidly internalised and the cytotoxic moiety (cys-mcMMAF) is released, leading to direct cell death. Indeed, preclinical studies demonstrated in vitro and in vivo cytotoxic activity against both myeloma cell lines and primary patient cells^4^.

We conducted a phase I, first-in-human, open-label study with dose escalation (part 1) and dose expansion (part 2), which assessed the safety, pharmacokinetics (PK) and preliminary clinical activity of GSK2857916 monotherapy in patients with relapsed/refractory MM. Results of the prespecified interim analysis^7^ indicated that GSK2857916, at the identified recommended phase II dose of 3.4 mg/kg, demonstrated favourable PK properties, was well tolerated and had good clinical activity in heavily pre-treated patients. Here, we report an update of the safety and efficacy results of part 2, following an additional 14 months of follow-up from the date of the interim analysis data cut.

## Materials and methods

### Study design

This multicentre, open-label, first-in-human, phase I study was conducted at nine centres in the USA, Canada, and the UK (BMA117159; NCT02064387). The study comprised two parts: part 1 was a dose-escalation phase that assessed the safety and tolerability of GSK2857916 to establish the recommended dose; part 2 confirmed the safety, tolerability, PK, and efficacy of GSK2857916 at the dose identified in part 1. Full methodological details of this study are reported in Trudel et al.^7^.

The study was conducted according to good clinical practice, was approved by appropriate ethics committees and institutional review boards at each study site and all patients provided written informed consent.

### Patients

Eligible adult (≥18 years of age) patients for part 2 had histologically or cytologically confirmed MM, Eastern Cooperative Oncology Group performance status 0 or 1, prior therapy with alkylators, proteasome inhibitors and immunomodulators, and were refractory to the last line of treatment (defined as progression on or within 60 days of completion of the last therapy), and measurable disease (defined as having at least one of the following: serum M-protein ≥0.5 g/dL, urine M-protein ≥200 mg/24 h, serum free light chain ≥5 mg/dL and abnormal serum free light-chain ratio [ <0.26 or >1.65], or plasmacytoma confirmed with biopsy). BCMA expression was not required for enrolment. Full inclusion and exclusion criteria are listed in Trudel et al.^7^.

### Endpoints

The full list of trial endpoints is provided in Trudel et al.^7^. Here we report an update on safety endpoints (primary), clinical activity (secondary) and the exploratory endpoints of progression-free survival, duration of response and time to response.

### Study treatment

GSK2857916 3.4 mg/kg was administered through 1-h intravenous infusions once every 3 weeks, for a maximum of 16 cycles; the dose was selected based on part 1 PK and tolerability results^7^. Steroid eye drops (prednisolone phosphate 1% or dexamethasone 0.1% four times per day for 4 days starting 1 day before each GSK2857916 dose) were used by all patients at the time of each infusion to mitigate corneal events, a known toxic effect of MMAF^8^.

### Study assessments

Full details on study assessments can be found in Trudel et al.^7^ and are summarised here. Patients were initially followed up for up to 3 months after the end of the treatment; the protocol was amended to follow up patients for up to 1 year after end of treatment. To assess the safety and tolerability of GSK2857916, adverse events (AEs) were recorded from the first dose until 30 days after the last dose. AEs that occurred within the first 21 days of treatment and for which association with study treatment could not be excluded were considered a dose-limiting toxicity. Specific criteria for dose-limiting toxicity are provided in Trudel et al.^7^.

Clinical activity of GSK2857916, measured as overall response rate, was assessed according to the International Myeloma Working Group uniform response criteria for MM^9^. Disease assessment was completed every 3 weeks or at the start of each treatment cycle until the final study visit.

### Data analysis

Demographics and safety data were analysed descriptively. Overall response was calculated with two-sided 95% exact confidence intervals (CIs). Progression-free survival, duration of response, and time to response were analysed using the Kaplan–Meier method. All patients who received at least one dose of GSK2857916 were included in the analyses.

### Data sharing statement

Anonymised individual participant data and study documents can be requested for further research from www.clinicalstudydatarequest.com.

## Results

### Patient population

**Fig. 1:**
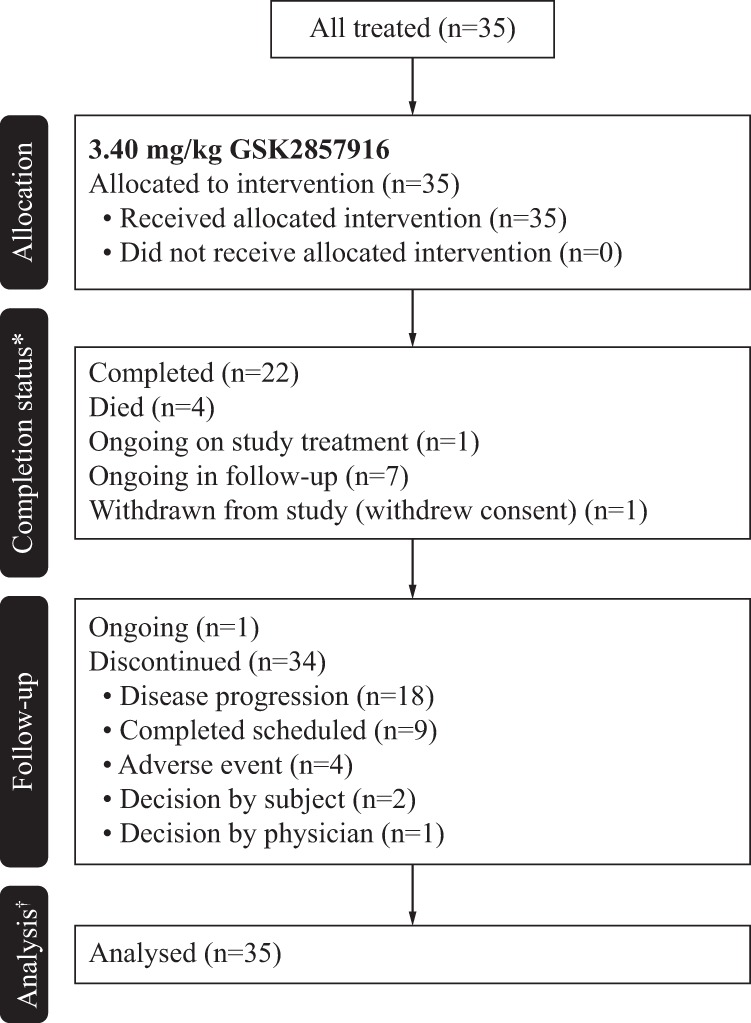
**Study profile.** *A completed subject is one who has completed at least one cycle of study treatment and an end of study visit without events causing them to withdraw or discontinue from the study; ^†^Analysed for safety and clinical activity of study intervention

Enrolment of patients for part 2 took place from August 9, 2016 to December 7, 2016; all patients recruited in 2017 were dosed at GSK2857916 2.5 mg/kg in part 1, an additional dosing level not included in the original dose escalation schedule but added later to further assess safety^7^. As of the data cut-off date of August 31, 2018, of the 35 patients treated in part 2, 22 completed the study, 7 are ongoing in follow-up, and 1 patient is continuing with study treatment (Fig. 1). All 35 patients were included in the analyses. Patient baseline demographics are shown in Table 1. Fourteen of the 35 patients had received >5 previous lines of therapy. The median duration of follow-up was 12.5 months (range 0.7–23.2).

**Table 1:** Patient baseline characteristics and disposition

Characteristic	*n* = 35
Age, median (range) years	60 (46–75)
Sex	
Men	17 (49%)
Women	18 (51%)
Disease stage at diagnosis^a^	
Stage I	19 (54%)
Stage II	6 (17%)
Stage III	4 (11%)
Unknown	6 (17%)
ECOG PS	
0	10 (29%)
1	25 (71%)
Myeloma light chain	
Kappa light chain	24 (69%)
Lambda light chain	11 (31%)
Myeloma immunoglobulin	
IgA	8 (23%)
IgG	22 (63%)
IgM	1 (3%)
Other	4 (11%)
Genetics, *n* (%)^b^	
del13	6 (17%)
del17p13	6 (17%)
t(11:14)	2 (6%)
t(4:14)	3 (9%)
t(14:16)	1 (3%)
1q21	3 (9%)
Other	14 (40%)
Missing	11 (31%)
Prior therapies	
1 line	1 (3%)
2 lines	2 (6%)
3 lines	7 (20%)
4 lines	5 (14%)
5 lines	6 (17%)
6 lines	3 (9%)
7 lines	2 (6%)
8 lines	3 (9%)
9 lines	2 (6%)
10 lines	2 (6%)
>10 lines	2 (6%)
Proteasome inhibitors	
Received	35 (100%)
Refractory	34 (97%)
Immunomodulatory drugs	
Received	35 (100%)
Refractory	33 (94%)
Pomalidomide	
Received	22 (63%)
Refractory	22 (63%)
Daratumumab	
Received	14 (40%)
Refractory^c^	14 (40%)
Carfilzomib	
Received	29 (83%)
Refractory	27 (77%)
Patient disposition	
Completed study	22 (63%)
Died	4 (11%)
Ongoing on study	8 (23%)
On treatment	1 (3%)
In follow-up	7 (20%)
Withdrawn from study	1 (3%)
Withdrew consent	1 (3%)
Lost to follow-up	0
Discontinued treatment	34 (97%)
Disease progression	18 (51%)
Completion of treatment	9 (26%)
Adverse event	4 (11%)
Investigator discretion	1 (3%)
Patient decision	2 (6%)

Data are *n* (%), unless otherwise specified

*ECOG PS* Eastern Cooperative Oncology Group performance status, *Ig* immunoglobulin

^a^Assessed using the International Staging System classification^9,^^26^

^b^Multiple categories per patient possible, resulting in a total that adds to more than 100%; assessed using fluorescence in situ hybridisation

^c^Thirteen (37%) of 35 patients had previous daratumumab treatment and were refractory to both immunomodulatory drugs and proteasome inhibitors

### Safety and tolerability

All patients experienced at least one AE, most commonly thrombocytopenia (22/35; 63%), blurred vision (18/35; 51%), and cough (14/35; 40%) (Table 2). Grade 3 or 4 AEs were reported in 29 (83%) patients, the most common of which were thrombocytopenia (grade 3, 9/35 [26%]; grade 4, 3/35 [9%]) and anaemia (grade 3, 6/35 [17%]); no grade 5 AEs were reported. Serious AEs (SAEs) were reported in 17/35 (49%) patients, most commonly pneumonia (3/35; 9%), lung infection (2/35; 6%), and infusion-related reactions (2/35; 6%). Seven (20%) patients experienced SAEs related to study treatment, most commonly infusion-related reactions (2/35; 6%). Four patients died during the study, all due to progression of MM.

**Table 2:** Summary of treatment-emergent adverse events

	Maximum grade, *n* (%)			
	Grade 1	Grade 2	Grade 3	Grade 4
Thrombocytopenia^a^	4 (11)	6 (17)	9 (26)	3 (9)
Blurred vision	2 (6)	15 (43)	1 (3)	0
Cough	11 (31)	3 (9)	0	0
Increased aspartate aminotransferase	8 (23)	3 (9)	2 (6)	0
Dry eye	6 (17)	6 (17)	1 (3)	0
Nausea	9 (26)	2 (6)	0	0
Anaemia	0	4 (11)	6 (17)	0
Diarrhoea	6 (17)	2 (6)	2 (6)	2 (6)
Photophobia	7 (20)	3 (9)	0	0
Pyrexia	6 (17)	4 (11)	0	0
Chills	7 (20)	2 (6)	0	0
Fatigue	2 (6)	6 (17)	0	0
Upper respiratory tract infection	5 (14)	3 (9)	0	0
Increased alanine aminotransferase	5 (14)	2 (6)	0	0
Back pain	3 (9)	2 (6)	2 (6)	0
Constipation	5 (14)	1 (3)	1 (3)	0
Increased γ-glutamyl transferase	2 (6)	3 (9)	2 (6)	0
Arthralgia	2 (6)	3 (9)	1 (3)	0
Increased blood alkaline phosphatase	6 (17)	0	0	0
Dyspnoea	4 (11)	1 (3)	1 (3)	0
Contusion	5 (14)	0	0	0
Decreased appetite	3 (9)	2 (6)	0	0
Headache	4 (11)	1 (3)	0	0
Sinusitis	0	5 (14)	0	0
Eye pain	3 (9)	0	1 (3)	0
Hypokalaemia	1 (3)	0	3 (9)	0
Infusion-related reaction	1 (3)	2 (6)	1 (3)	0
Lung infection	0	1 (3)	3 (9)	0
Pneumonia	0	1 (3)	3 (9)	0
Urinary tract infection	0	3 (9)	1 (3)	0
Hypertension	1 (3)	1 (3)	1 (3)	0
Keratitis	0	1 (3)	2 (6)	0
Decreased neutrophil count	0	0	3 (9)	0
Haematuria	0	1 (3)	1 (3)	0
Neutropenia	0	0	1 (3)	1 (3)
Rib fracture	1 (3)	0	1 (3)	0
Toothache	0	1 (3)	1 (3)	0
Appendicitis	0	0	1 (3)	0
Bacteraemia	0	0	0	1 (3)
Increased blood lactate dehydrogenase	0	0	1 (3)	0
Cataract	0	0	1 (3)	0
Cholecystitis acute	0	0	0	1 (3)
Cholecystitis infective	0	0	0	1 (3)
Deep vein thrombosis	0	0	1 (3)	0
Encephalopathy	0	0	1 (3)	0
Fall	0	0	1 (3)	0
Febrile neutropenia	0	0	1 (3)	0
Gastroenteritis	0	0	1 (3)	0
Humerus fracture	0	0	1 (3)	0
Lower respiratory tract infection	0	0	1 (3)	0
Pericardial effusion	0	0	0	1 (3)
Pneumonia haemophilus	0	0	1 (3)	0
Respiratory tract infection	0	0	1 (3)	0
Retinal detachment	0	0	1 (3)	0
Salmonellosis	0	0	1 (3)	0
Syncope	0	0	1 (3)	0
Abnormal visual acuity tests	0	0	1 (3)	0

All adverse events of grades 3 and 4 are shown, and adverse events of grades 1 and 2 that occurred in 10% or more of patients (*n* = 35). No grade 5 events occurred

^a^Grouped term includes thrombocytopenia and decrease in platelet count

Four (11%) patients had AEs that led to permanent discontinuation of study treatment, each due to thrombocytopenia, keratopathy, fatigue and cough, and increased alanine aminotransferase, aspartate aminotransferase, and blood creatine phosphokinase. Overall, 23 (66%) had AEs that caused dose reductions, most commonly blurred vision (12/35; 34%) and thrombocytopenia (6/35; 17%); 25 (71%) had AEs that led to dose interruptions or delays, most commonly blurred vision (14/35; 40%), followed by thrombocytopenia (5/35; 14%), and keratitis, photophobia, and pneumonia (each reported by 3 [9%] patients).

AEs of special interest included infusion-related reactions, thrombocytopenia, and corneal events. To fully assess the incidence and severity of infusion-related reactions, medications to prevent such reactions were not permitted for the first infusion, but were allowed with subsequent infusions. Ten patients (29%) had infusion-related reactions, most of which were grade 1 or 2; all occurred with the first dose of GSK2857916. The median time of onset of thrombocytopenia was 7.5 days (range 5–365) and the median duration for patients with a resolution date (*n* = 10) was 8 days (range 6–267). One (6%) patient discontinued treatment because of grade 3 thrombocytopenia and seven (20%) required dose reductions or delays/interruptions due to thrombocytopenia. Corneal events were reported in 24 (69%) of patients, most commonly blurred vision (18/35; 51%), dry eye (13/35; 37%) and photophobia (10/35; 29%). Most patients experienced grade 1 or 2 corneal events (19/35; 54%); 5 (14%) patients had grade 3 events. The median duration of corneal events for patients with a resolution date (*n* = 16) was 35 days (range 5–442). Corneal events led to dose reduction in 16 (46%) patients, and dose interruptions or delays in 17 (49%) patients.

### Clinical efficacy

**Fig. 2:**
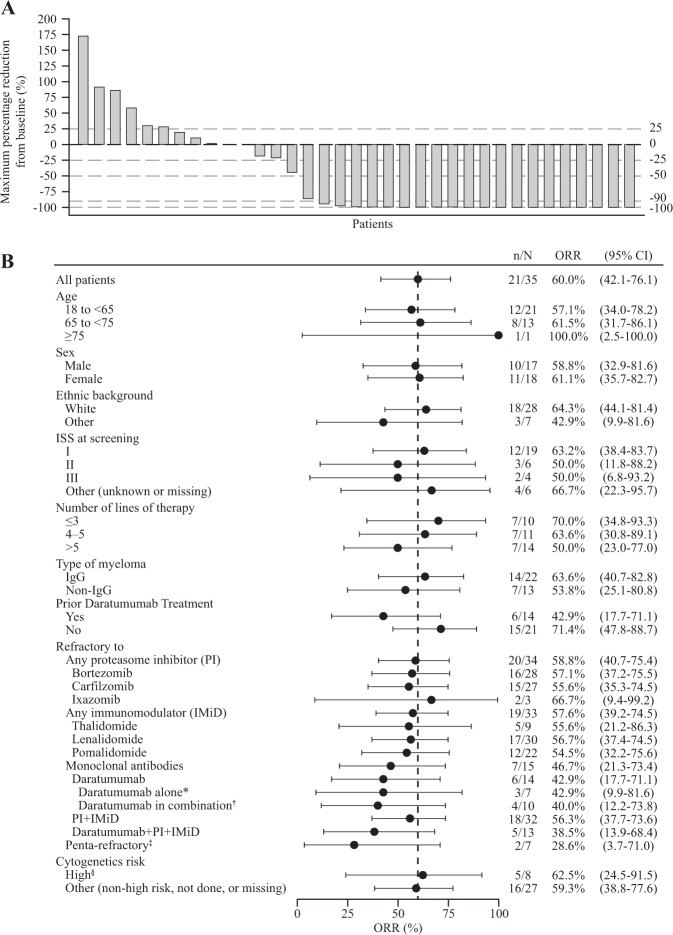
**Best responses to GSK2857916. a** Maximum percentage change from baseline in M-protein or free light chain. For patients with measurable serum M-protein, serum concentration is shown; for patients with urine M-protein measurements, urine concentrations are shown; and for patients with no available serum or urine M-protein measurements, free light-chain concentrations are shown. **b** Forest plot of overall response rates by patient subgroup. *Defined as prior cyclophosphamide (CTX) regimen with daratumumab as the only drug in the regimen; ^†^defined as prior CTX regimen with daratumumab and other drugs in the regimen; ^‡^defined as refractory to bortezomib, carfilzomib, lenalidomide, pomalidomide and daratumumab; ^§^a patient is considered as high risk if the subject has any of the following cytogenetics: t(4;14), del17p and t(14;16)

**Fig. 3:**
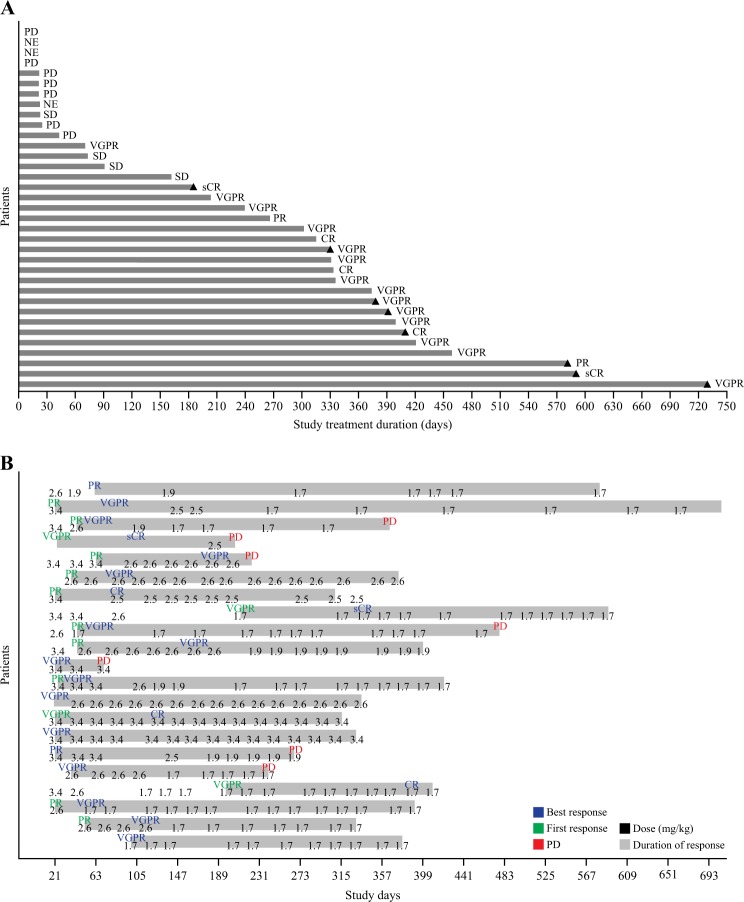
**Response duration and effect of dose modifications.** **a** Duration of study treatment by response. Treatment duration counts the time difference between first dose date and final dose date without accounting for dosing interruptions. Triangles indicate patients remaining on the study. **b** Dose modifications in responding patients. For each of the 21 responding patients, initial response (partial response or better) is indicated in green, best response in blue, and progressive disease in red. The numbers indicate dose (mg/kg) for each infusion. CR, complete response; NE, not evaluable; PD, progressive disease; PR, partial response; sCR, stringent complete response; SD, stable disease; VGPR, very good partial response

Twenty-one patients had a confirmed response of partial response or better (60.0%; 95% CI 42.1–76.1), with two (6%) patients achieving a stringent complete response, and an additional three (9%) achieving a complete response, 14 (40%) achieving a very good partial response, and two (6%) achieving a partial response (Figs. 2a, 3a). Overall response rates grouped by baseline characteristics are shown in Fig. 2b. Of the 32 patients refractory to both immunomodulators and proteasome inhibitors, confirmed overall response was achieved in 18 patients (56.3%; 95% CI 37.7–73.6). Of the 21 patients without prior daratumumab treatment, 15 achieved a confirmed overall response (71.4%; 95% CI 47.8–88.7); of the 13 patients with prior daratumumab treatment and refractory to both immunomodulators and proteasome inhibitors, five had a confirmed overall response (38.5%; 95% CI 13.9–68.4).

**Fig. 4:**
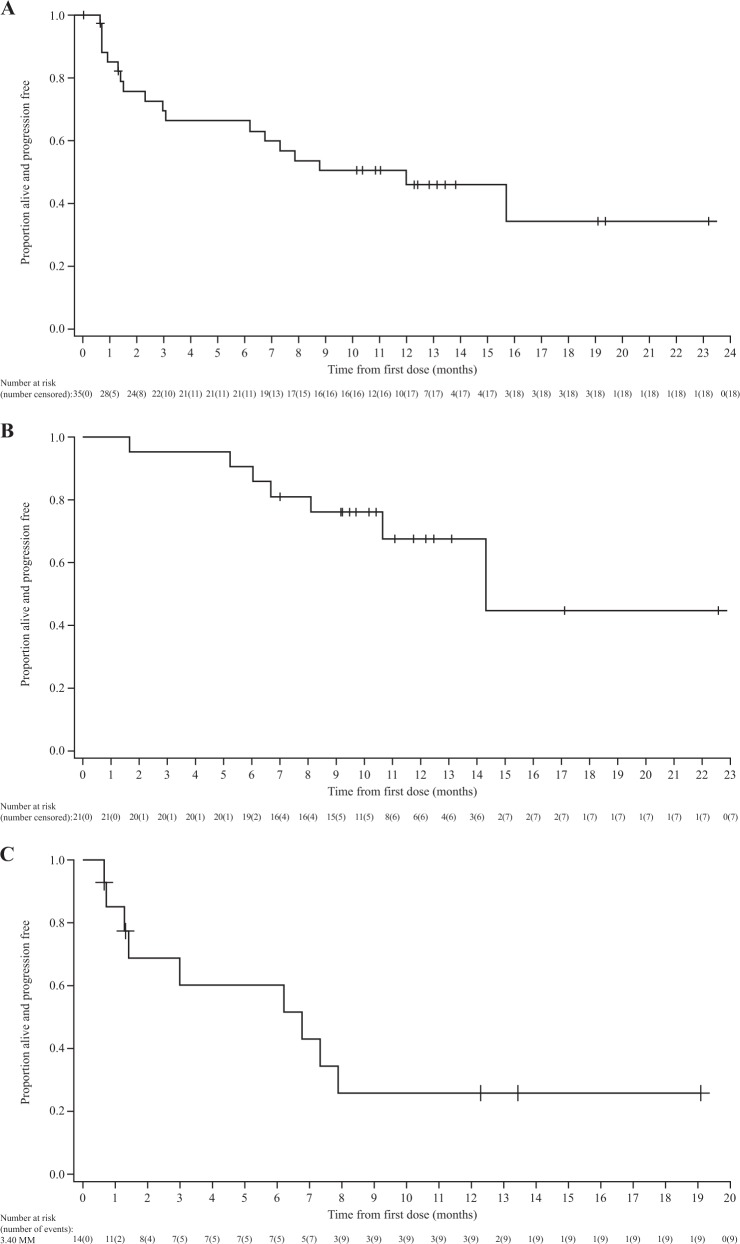
Kaplan–Meier curves for **a** progression-free survival and **b** duration of response in the overall population, and **c** progression-free survival in patients with prior daratumumab

The median time to first response was 1.2 months (95% CI 0.7–1.4); responses were maintained and generally deepened over time (Fig. 3a, b). The median progression-free survival was 12.0 months (95% CI 3.1–not estimable) (Fig. 4a) and the median duration of response was 14.3 months (95% CI 10.6–not estimable) (Fig. 4b). In patients refractory to both immunomodulators and proteasome inhibitors, median progression-free survival was 7.9 months (95% CI 2.3–not estimable); in patients without prior daratumumab treatment, median progression-free survival was 15.7 months (95% CI 2.3–not estimable). In patients with prior daratumumab treatment, median progression-free survival was 6.8 months (95% CI 1.3–not estimable) (Fig. 4c); and in those with prior daratumumab treatment and refractory to, immunomodulators, and proteasome inhibitors, median progression-free survival was 6.2 months (95% CI 0.7–7.9).

## Discussion

This update of a phase I, first-in-human study demonstrates the high single-agent activity of anti-BCMA GSK2857916 therapy in patients with relapsed or refractory MM. With an additional 14 months of follow-up, we observed more complete responses and a longer progression-free survival compared with interim analyses, showing rapid, deep, and durable responses with GSK2857916. The interim analyses for the study (data cut-off date June 26, 2017) at a median follow-up of 12.5 months indicated a confirmed overall response in 21 (60.0%) of the 35 patients in part 2 of the trial, including one stringent complete response and two complete responses. After over a year of additional follow-up, we found an identical overall response rate in these patients, but responses had deepened over time and the number of stringent complete responses and complete responses had increased from one to two, and from two to three, respectively. Further, our analyses have provided an update on the progression-free survival and duration of response with GSK2857916: the median progression-free survival, previously at 7.9 months, is now estimated at 12.0 months (95% CI 3.1–not estimable) with an additional follow-up of 14 months, and the duration of response, not previously estimable at the interim analyses, is estimated at 14.3 months. Overall, these results show a sustained overall response rate, which has deepened over time. Importantly, the median duration of response and median progression-free survival are considerably longer than initially reported at the interim analysis^7^.

Treatment options for MM have increased in recent years and the introduction of novel proteasome inhibitors and immunomodulatory drugs is significantly associated with prolonged survival in patients with MM^10,11^. However, outcomes remain poor for patients with relapsed and refractory disease, with those refractory to both proteasome inhibitors and immunomodulatory drugs having an estimated survival of only 13 months^2^. Subgroup analyses in our study revealed an overall response rate of 56% in patients refractory to both proteasome inhibitors and immunomodulators, similar to the rate achieved in the overall population, suggesting GSK2857916 may be a promising treatment option in heavily pre-treated refractory patients.

In 2015, daratumumab, an anti-CD38 monoclonal antibody, was approved for patients who have received at least three prior lines of therapy or who are double refractory to a proteasome inhibitor and an immunomodulator, and is currently a recommended therapy option in patients with relapsed/refractory disease^12,13^. Still, with a reported overall response rate of 36%^14^, most patients fail to respond to single-agent daratumumab, and the outcome of patients following failure of daratumumab therapy is poor, with a reported median overall survival of 5.3–8.6 months in two recent retrospective studies^15,16^. Furthermore, patients refractory to anti-CD38 antibodies become increasingly less responsive with subsequent lines of therapy^15,16^.

In this study, although the numbers are small, subgroup analysis found an overall response rate of 71% in those without prior daratumumab versus 42.9% with a progression-free survival of 6.8 months in patients refractory to daratumumab. While the response rate is lower in the latter population, it is still an encouraging response for a population of patients who typically respond poorly with any treatments following daratumumab failure. Indeed, a study of patients refractory to anti-CD38 antibody therapies (of which 93.5% were daratumumab-refractory) found the overall response rate of the first treatment regimen after progression with anti-CD38 therapy was 31%, with a median progression-free survival of just 3.4 months^17^. In particular, use of elotuzumab-based therapies after failure of anti-CD38 therapy led to a low overall response rate of 21%, while the addition of an immunomodulatory drug to daratumumab treatment resulted in a higher overall response rate (37%) and slightly longer progression-free survival (4.5 months). Therefore, the results of our study suggest GSK2857916 may help to redefine treatment expectations in heavily pre-treated patients refractory to daratumumab. Further studies are required to identify a possible reason for the difference in response rate between prior or no prior daratumumab subgroups, such as analysing the number of prior lines of therapy, cytogenetic risk, and expression of soluble or surface expression of BCMA between groups. Indeed, it is likely that the patients in the study who had received daratumumab had MM for longer and had more refractory disease with more clonal/subclonal evolution.

Immunotherapy is a rapidly developing field in MM. Currently the only antibody-based therapies to be approved are daratumumab and elotuzumab (anti-SLAMF7); in contrast to the target antigens of these therapies, BCMA is specifically expressed on normal and malignant plasma cells, but no other cells^6,18^. Two other antibody–drug conjugates that target BCMA are also in development, HDP-1 and MEDI2228, and have demonstrated anti-MM activity in preclinical studies^19,20^. Furthermore, anti-BCMA bi-specific T cell engagers and chimeric antigen receptor (CAR) T cells are being developed^20,21^, supporting the use of BCMA as a target in novel therapies. The clinical activity of GSK2857916 compares favourably with those described previously for CAR T cells targeting BCMA. Phase I studies of BCMA CAR T cells have demonstrated response rates of 64% to 96% at effective doses (>10^8^ CAR-positive cells)^22–25^, and progression-free survival of 11.8 months has reported for bb2121^22,26^. However, GSK2857916 does not have the risks of cytokine release syndrome or neurotoxicity present with BCMA CAR T cells^22,23,25^, and it offers the added advantage of scalability and feasibility, including outpatient administration, over CAR T cells.

Our results also demonstrate an acceptable safety profile with GSK2857916. The most commonly reported AEs were cough, increased aspartate aminotransferase, and nausea, all of which were mostly mild or moderate (grade 1 or 2), in addition to corneal events and thrombocytopenia, which are consistent with the known toxic effects of other MMAF-linked antibody–drug conjugates^8^ and were found to be manageable. Studies are ongoing to further characterise and understand the corneal events with GSK2857916 and future studies will also investigate the benefits of convenient cooling eye masks and increasing the duration of steroid eye drop use from 4 to 7 days to mitigate corneal events. Importantly, no new safety signals were identified since the interim analyses^7^.

In conclusion, GSK2857916 demonstrated a rapid, deep, and durable clinical response with a significant progression-free survival in a heavily pre-treated population. New subgroup analyses indicate a benefit for patients refractory to proteasome inhibitors and immunomodulatory drugs, and for those with prior daratumumab treatment. The favourable safety profile and clinical activity of GSK2857916 monotherapy support progress into larger later-phase trials. Future studies may also investigate the use of GSK2857916 as a key component for combination with other therapies and in other MM populations. Overall, our results suggest that GSK2857916 is a promising therapy for patients with relapsed and refractory MM, including those in whom all other standard and available therapies have failed.
